# Using Open Geographic Data to Generate Natural Language Descriptions for Hydrological Sensor Networks

**DOI:** 10.3390/s150716009

**Published:** 2015-07-03

**Authors:** Martin Molina, Javier Sanchez-Soriano, Oscar Corcho

**Affiliations:** Department of Artificial Intelligence, Technical University of Madrid, Campus de Montegancedo S/N, 28660 Boadilla del Monte, Madrid, Spain; E-Mails: jsanchez@fi.upm.es (J.S.-S.); ocorcho@fi.upm.es (O.C.)

**Keywords:** sensor network, natural language generation, open geographic data

## Abstract

Providing descriptions of isolated sensors and sensor networks in natural language, understandable by the general public, is useful to help users find relevant sensors and analyze sensor data. In this paper, we discuss the feasibility of using geographic knowledge from public databases available on the Web (such as OpenStreetMap, Geonames, or DBpedia) to automatically construct such descriptions. We present a general method that uses such information to generate sensor descriptions in natural language. The results of the evaluation of our method in a hydrologic national sensor network showed that this approach is feasible and capable of generating adequate sensor descriptions with a lower development effort compared to other approaches. In the paper we also analyze certain problems that we found in public databases (e.g., heterogeneity, non-standard use of labels, or rigid search methods) and their impact in the generation of sensor descriptions.

## 1. Introduction

Sensor networks are usually part of infrastructures for the management of complex dynamic systems. The data collected by sensors can help users in decision-making tasks (e.g., road networks for traffic surveillance, river channels for water management). These networks can include thousands of sensors that periodically (every hour, every 15 min, *etc.*) measure physical magnitudes, producing large amounts of quantitative data with both spatial and temporal references.

Several tools and middleware exist for the provision of data access mechanisms to sensor data (e.g., GSN [1], SOS-compliant services [2], Xively [3], *etc.*). Some of these tools can also provide interpretations of data observed by sensors by translating such data into more natural and intuitive descriptions understandable by the general public.

In the case of geographically distributed networks, with hundreds or thousands of sensors and different types of measurements, it is also important for end users to have a textual description that refers to the spatial location of events as understood by them. For example, in the hydrologic domain, sentences like “there is heavy rain in the South of Spain” or “the water level in the Ebro River at Ascó has decreased” are more useful than “the precipitation is 34 mm/h in the area associated to bounding box X”.

In this paper, we describe how such textual descriptions can be generated for sensors, with a focus on geographic references. This problem is related to a more general problem called reference expression generation [4], as defined by the natural language generation community. The spatial nature of geographic sensor networks makes this problem different from other general approaches [5]. For example, it is necessary to use detailed and diverse geographic knowledge (such as coordinates, places, distances, natural formations, *etc.*), which is not always easy to acquire and maintain up-to-date. However, the increasing availability of geographic information on the Web (e.g., OpenStreetMap, Geonames, *etc.*) in recent years is a potential alternative solution to this problem.

In this paper, we describe the results of our recent research in this area. We discuss the feasibility of using geographic knowledge from open geographic databases available on the Web to automatically construct sensor descriptions in natural language. We present a method following this approach that we evaluated using a national hydrologic sensor network with thousands of sensors in Spain. In general, the evaluation results show that this approach generates acceptable textual descriptions. However, the current versions of existing online geographic databases still present some problems when generating appropriate textual descriptions (e.g., heterogeneity, non-standard use of labels, or rigid search methods). In this paper, we also analyze these problems and propose future solutions to improve the performance of our sensor network textual generation method.

## 2. Problem Description

In this section we describe the problem of generating textual descriptions for geographically distributed sensors, starting from quantitative sensor data.

### 2.1 Motivation and Context

In general, the data collected by sensor networks for the management of dynamic systems are potentially useful not only to domain experts but also to wide audiences with a great diversity of users. For example, sensor networks for monitoring water in natural environments can help users such as municipalities, civil protection agencies, consultants, insurance companies, scientist researchers, journalists, educators, and general citizens. The information can help make management decisions (e.g., about agriculture, hydroelectric energy production, flood risk, or climate change) and can also contribute to increasing the awareness of the general public about important issues (e.g., the limited availability of natural resources such as water to promote their better use with sustainable practices).

**Figure 1:**
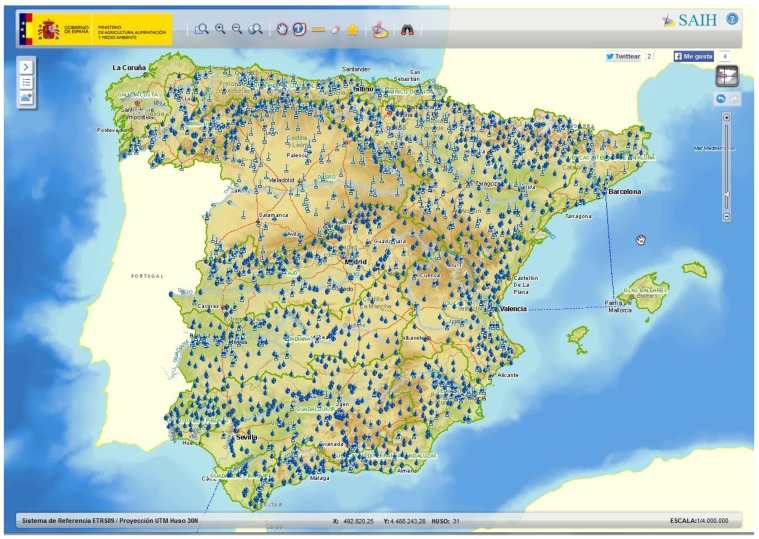
Geographic distribution of the national hydrological sensor network SAIH (part of the network shown by the website of the Ministry of Environment of Spain).

However, it is not always easy for such communities of users to access and understand data from sensor networks. Datasets are normally represented using technical terminology and professional jargon (sensor devices, code identifiers, physical magnitudes, *etc.*), which may be unfamiliar to certain users. For example, in Spain, the SAIH system (*Sistema Automático de Información Hidrológica*) is a national hydrologic sensor network that has been operating for more than 20 years [6] (Figure 1). This network collects real-time hydrological data (water flows, water levels, rainfall, *etc.*) recorded by thousands of sensors at different locations. The information collected by the SAIH network is useful for different tasks, such as early warnings for floods or water management. Sensors in this network are identified using a set of conventions established by technical operators. For example, the sensor C001L85PQUIN refers to a sensor that measures the accumulated rainfall in hourly intervals at a certain location of the Ebro basin. These types of references can be effectively used by domain experts. However, for the general public, these identifiers and code lists represent an important language barrier that prevents non-experts from an easy understanding of data.

In addition, using datasets directly requires familiarity with computer technology, such as data formats and software tools, that also presents difficulties to general users who are interested in the data. Some software applications help users interpret these datasets with graphical browsers and visualization systems. However, these solutions provide limited support to the general public because such technology normally assumes familiarity with domain knowledge to interpret data and graphic visualizations and requires that users take the initiative for what to search.

### 2.2. Generation of Natural Language Descriptions

To facilitate access to sensor data to large communities of users, a possible solution is using data-to-text systems [7,8,9]. The basic idea is to use computational methods that get data from sensor networks and automatically generate textual descriptions in natural language that help users to understand the meaning of the data with interpretations and explanations.

An example of the data-to-text solution was implemented for the VSAIH system (*Vigilancia Hidrológica Automática – Sistema de Información SAIH*) [10]. This system analyzes data from the SAIH hydrological national sensor network and follows a journalistic style to generate news stories for the general public. VSAIH automatically generates news mainly related to descriptive analytics (*what happened?*) and also diagnostic analytics (*why did it happen?*) about flood risk, availability of water resources, and sensor faults (see [11] for more details).

**Figure 2:**
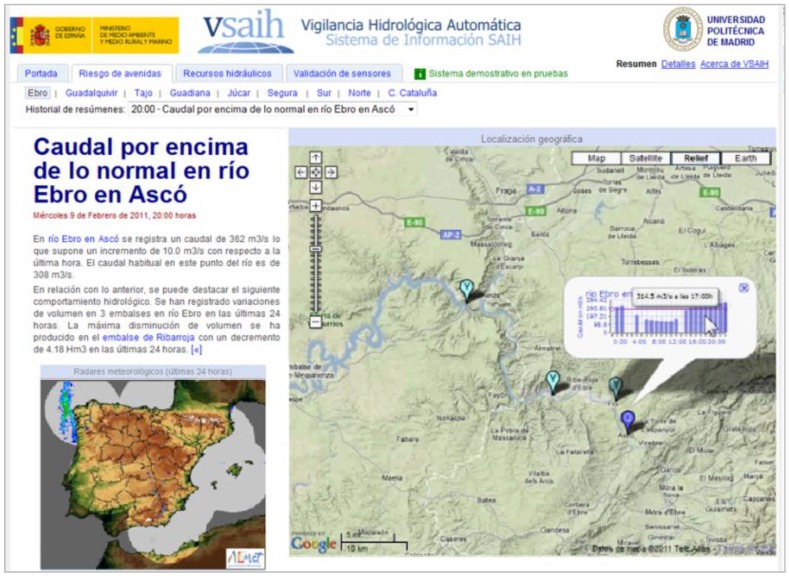
Example of a presentation generated by the VSAIH application.

**Figure 3:**
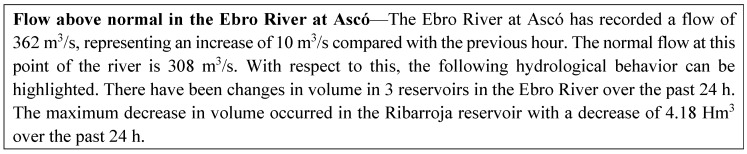
Generated text describing a hydrologic situation (translated from Figure 2).

Figure 2 shows an example of a presentation generated by the VSAIH system (in Spanish) and Figure 3 shows the text translation. The presentation includes a journalistic text description with a headline and body text along with some interactive graphics (maps, charts, animations, *etc.*). The practical evaluation of VSAIH [11] showed that these type of descriptions are able to facilitate decision-making and save critical time in special emergency scenarios.

In general, beyond the type of presentation generated for VSAIH, the generation of natural language descriptions for sensor networks can also provide more flexibility in combination with text-to-speech converters to be used in multimodal communication contexts (e.g., speech communication with sensor networks, or informing visually impaired people with audio messages about sensor data, *etc.*).

The generation of such textual descriptions requires that the specific problem of referring expression generation (REG) for geographic locations be solved [4] (for example, the generation of references such as: “the Ebro River at Ascó” or “the Ribarroja River”). In general, the methods for REG have evolved since the early works [12,13,14] to more recent frameworks such as graph search [15] or constraint satisfaction [16].

However, the problem of generating text descriptions for geographically distributed sensors is different from other general approaches [5]. One of the main problems is that sensor datasets contain little explicit geographic information (typically, only geographic coordinates) and the natural language generation process needs to use a large amount of geographic knowledge to generate the descriptions. For example, it is necessary to know names of different geographic entities (populated places, administrative regions, forests, rivers, basins, mountains, historical places, *etc.*) together with diverse spatial relations (geographic locations, spatial shapes, *etc.*) to generate useful descriptions.

Domain experts (e.g., experts in the SAIH sensor network in Spain) generate text descriptions using geographical references based on their domain knowledge of the spatial area. The variety of potential geographic references used by experts (e.g., names of populated places, natural formations, historical monuments, *etc.*) shows that this is not a trivial problem. For example, we may think that it is sufficient if we select the populated place nearest to the geographic location of the sensor (given by its latitude and longitude). However, some sensors can be located at certain unpopulated places (forests, mountains, parks, *etc.*), or another populated place may be more significant (e.g., it has higher number of inhabitants or it is better known for historical reasons), or the populated place is not relevant enough compared to other references (monuments, constructions, *etc.*). In those cases, another name is used, corresponding to another populated place, such as a monument, a construction, *etc.*

In a previous work [17,18], we showed a solution for this problem that we applied to the VSAIH system. This solution was developed using a geographic domain knowledge base acquired from hydrological experts. However, this solution has two limitations: (1) the construction of such a knowledge base requires an important acquisition effort and needs to be maintained up-to-date, and (2) it is limited to a specific geographic area. In this paper we describe a new approach that tries to solve these two limitations using geographic open data from the available large-scale online datasets on the Web. The following sections describe this type of open data and, then, how we used it to generate textual descriptions for sensor networks.

## 3. Open Geographic Data

Open geographic data is a potential source of information that can help automatically construct sensor descriptions. In the last years, several efforts have been made for the provision of large-scale datasets on the Web under open licenses. Examples of such datasets include OpenStreetMap, Geonames, DBpedia, and many others inside and outside the geographical domain (e.g., the datasets available in the Linked Open Data cloud). Some authors [19,20] have successfully used this type of dataset in geographic problems (route directions).

A good number of these datasets have been made available following the Linked Data principles [21] and may or may not be registered in the aforementioned Linked Open Data (LOD) cloud. These principles can be summarized as making data available on the Web using a pure Web approach, defining URIs (Uniform Resource Identifiers) for things and making those URIs de-referenceable. The amount of LOD-compliant geographic data sources is quite significant, with worldwide approaches such as Geonames, and national and regional ones such as the GeoLinked Data initiative for Spain [22]. This open data movement is also being adopted by cities, which are incorporating their datasets into open data portals and application programming interfaces (APIs), providing all sorts of geographical information. That may be useful for more fine-grained tasks.

In the problem presented in this paper, as was mentioned previously, it is necessary to know geographical information such as the names of different geographic entities (populated places, administrative regions, forests, rivers, basins, mountains, historical places, *etc.*) together with diverse spatial relations (geographic coordinates, spatial shapes, *etc.*). It is also important to have data related to the relevance of spatial locations (e.g., number of inhabitants, number of photographs to evaluate the popularity of certain landmarks, *etc.*). According to these needs, we identified the following online databases with geographic data that can be used in our problem:

- *Geonames*: Geonames is a geographical name server that includes more than 7.5 million locations. Given a set of World Geodetic System 1984 (WGS84) geographical coordinates, it provides a suitable name for the specified location. The name is selected based on different features (populated place, road, hydrographic, *etc.*). Geonames contains place names in numerous languages. The coordinates are expressed in the standard WGS84. Geonames has a Creative Commons License (Attribution 3.0 Unported CC BY 3.0 license).
- *OpenStreetMap*: OpenStreetMap (OSM) is a geographic data server that allows free access to the full map dataset. This data can be downloaded in full or can be accessed using an API. OpenStreetMap has been created by thousands of volunteers, such as Wikipedia (in the geographical context, this is normally known as Volunteered Geographical Information). OpenStreetMap has the Open Database License (ODbL).
- *DBPedia*: The DBpedia project extracts structured information from Wikipedia and makes this information accessible on the Web under the terms of the Creative Commons Attribution-ShareAlike 3.0 License and the GNU Free Documentation License. DBpedia allows users to query relationships and properties associated with Wikipedia resources, including links to other related datasets.
- *Panoramio*: Panoramio is a geolocation-oriented photo-sharing website. Accepted photos uploaded to the site can be accessed as a layer in Google Earth and Google Maps, with new photos being added at the end of every month. The site’s goal is to allow Google Earth users to learn more about a given area by viewing the photos that other users have taken at that place. This database was selected to have information about the popularity of certain geographic locations. It was an open database when it was used in our experiments, although it is not freely available anymore.

## 4. The Method

**Figure 4:**
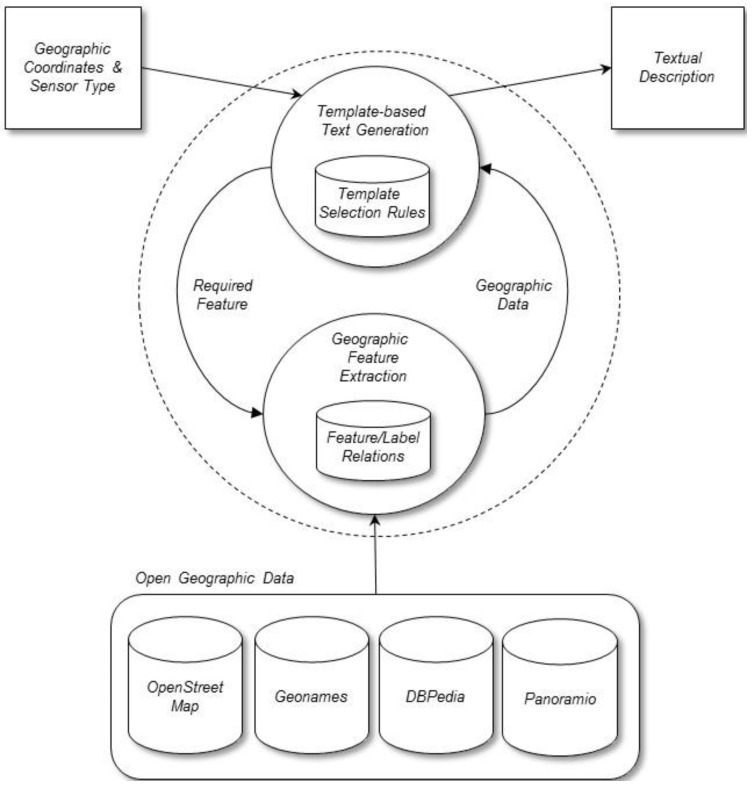
Main components of the method.

This section describes the method that we designed to generate textual descriptions for sensors using open geographic data (Figure 4). The method takes the geographical coordinates (latitude and longitude) of each sensor location and the sensor type, *i.e.*, the type of physical quantity measured by each sensor (e.g., water level, rainfall, flow, *etc.*), as input. The method also takes input data from the selected geographic databases (OpenStreetMap, Geonames, DBpedia, and Panoramio). As output, the method generates text descriptions that identify the geographical locations of sensors in natural language, for example: “the Ebro River at Ascó” or “the Ribarroja reservoir”. To construct the descriptions, our method performs two main processes: (1) template-based text generation and (2) geographic features extraction. The next sections describe these two processes in more detail.

### 4.1. Template-Based Text Generation

The first process follows a general heuristic approach using text templates. The text templates define patterns of natural language descriptions and they are selected and instantiated according to specific strategies formulated with a set of production rules. The rules provide flexibility to represent the criteria used by domain experts to create names for sensors. The rules help to generate the description, taking into account the physical quantity measured by the sensor, some geographic features, and other criteria established by domain experts. The general format of such rules is the following:

*SensorType*(*x*, *y*) ˄ *C*_1_ ˄ … ˄ *C_n_* → *Template*(*x, y*)

On the left-hand side, the rules include the logic predicate *SensorType*(*x*, *y*), *i.e.*, the type of sensor *x* is *y*, together with a set of conditions *C*_i_. To formulate the conditions, we use the logic predicate *Feature*(*x*, *y*, *z*), *i.e.*, for the sensor *x*, the geographic feature *y* has the value *z*, and comparison operators (e.g., equal to, greater than, less than, *etc.*) to establish conditions for the values of features. On the right-hand side, the rules include the logic predicate *Template*(*x*, *y*) that means that the textual description of sensor *x* is *y*. The representation of *y* is a list of text strings (for example (“river Ebro”, “at”, “Ascó”). The concatenation of these strings is the text description.

In the hydrologic domain, our method includes a small set of rules (20 rules) with their corresponding text templates that were obtained manually by analyzing text examples for hydrologic sensors written by hydrologic experts. For example, one of the strategies followed by experts is the following: if the sensor measures the river water flow, the text description should refer to the name of the river, but, if the sensor measures rain, the geographic reference does not include the name of the river. An example of the rule is:

*SensorType*(*s*, *WaterFlow*) ˄ *Feature*(*s*, *River*, *x*) ˄ *Feature*(*s*, *PopulatedPlace*, *y*) → *Template*(*s*, [*x*, “ *at* ”, *y*])

According to this rule, if the type of sensor *s* is *WaterFlow* and the feature *River* has the value *x* and the feature *PopulatedPlace* has the value *y*, then the text description for sensor *s* is the concatenation of the strings: *x*, “at ”, and *y* (for example the list (“River Ebro”, “at ”, “Ascó”)). The text description corresponding to a pluviometer is obtained using the following set of rules:

*SensorType*(*s*, *Pluviometer*) ˄ *Feature*(*s*, *Reservoir*, *x*) → *Template*(*s*, [*x*])
*SensorType*(*s*, *Pluviometer*) ˄ *Feature*(*s*, *HistoricPlace*, *x*) → *Template*(*s*, [*x*])
*SensorType*(*s*, *Pluviometer*) ˄ *Feature*(*s*, *Forest*, *x*) → *Template*(*s*, [*x*])
*SensorType*(*s*, *Pluviometer*) ˄ *Feature*(*s*, *MountainArea*, *x*) → *Template*(*s*, [*x*])
*SensorType*(*s*, *Pluviometer*) ˄ *Feature*(*s*, *Municipality*, *x*) → *Template*(*s*, [*x*])
*SensorType*(*s*, *Pluviometer*) ˄ *Feature*(*s*, *PopulatedPlace*, *x*) → *Template*(*s*, [*x*])

These rules help to identify the most appropriate geographic reference for the sensor location. The rules express that the textual description of a pluviometer is generated as follows: (1) if there is a reservoir at the sensor location we first use the name of the reservoir (feature Reservoir), (2) if there is no reservoir, but there is a historic place at the sensor location, we then use the name of the historic place (feature HistoricPlace), *etc.* As a control strategy, the rules are processed in the same order that they are written.

### 4.2. Geographic Feature Extraction

The second process of our method extracts the geographic features requested by the template-based text generation process using open geographic databases. For this purpose, this process uses an internal database that relates geographic features with specific labels to search in geographic databases. Table 1 shows the content of such database for the case of OpenStreetMap. For example, the geographic feature River can be found using six labels in OpenStreetMap (RIVER, STREAM, CANAL, *etc.*).

**Table 1:** Example of labels used in OpenStreetMap to select geographic features.

Geographic Feature	Labels in OpenStreetMap
*River*	RIVER, STREAM, CANAL, DRAIN, WATER, RIVERBANK
*PopulatedPlace*	TOWN, CITY, POPULATION
*MountainArea*	PEAK
*Waterwell*	WATERWELL
*Fountain*	DRINKINGWATER, SPRING
*Waterworks*	WASTEWATERPLANT, WATERWORKS, RESERVOIRCOVERED, WATERTOWER
*Reservoir*	DAM, RESERVOIR, WATER
*Forest*	FOREST, SCRUB, HEATH, WOOD
*HistoricPlace*	HISTORICPLACE

The geographic features of a sensor are obtained dynamically from the open geographic databases according to the following strategy:

1. *OpenStreetMap*: Initially, a set of candidate labels are obtained using OpenStreetMap. For this purpose, a bounding box around the geographic point of the sensor is used to formulate a query to OpenStreetMap. All the labels inside such a box are returned by OpenStreetMap. Then, our method searches for the required geographic feature (e.g., River) using the labels of Table 2 (e.g., RIVER, STREAM, CANAL, *etc.*). The bounding box is defined by four parameters: *X*_1_ minimum longitude, *y*_1_ minimum latitude, *x*_2_ maximum longitude, and *y*_2_ maximum latitude. If the geographic coordinates of the sensor location are *x* (longitude) and *y* (latitude), the parameters of the bounding box are obtained as follows: *X*_1_ = *x* – *k*_1_, *y*_1_ = *y* – *k*_1_, *x*_2_ = *x* + *k*_1_, and *y*_2_ = *y* + *k*_1_, where *k*_1_ is an adjustable parameter. For the tests presented in this paper we used the value *k*_1_ = 0.01 degrees for pluviometers and water flow sensors, and *k*_1_ = 0.018° for water level sensors for reservoirs. The values for *k*_1_ were estimated experimentally according to two goals: (1) obtain good candidate geographic references taking into account acceptable spatial distances between the geographic point and close spatial references (e.g., populated places) and the size of certain geographic features (e.g., reservoirs); and (2) keep the size of the bounding box as small as possible to avoid an excessive amount of data returned from OpenStreetMap.
2. *Geonames*: A search in Geonames is done to obtain new geographical information when there is no information available in OpenStreetMap for the features PopulatedPlace and MountainArea. For the feature PopulatedPlace, we use the label ADMINISTRATIVE in Geonames to find the populated place nearest to the sensor location. In this case, Geonames returns a list of places and the first place is selected as the value of the geographic feature. For the feature MountainArea we use the label HYPSOGRAPHIC.
3. *DBpedia*: Our method consults DBPedia to get the feature number of inhabitants of populated places. Our method selects places when the number of inhabitants *N* satisfies *N* > *k*_2_ (where *k*_2_ is a calibration parameter). For the experimental tests presented in this paper we used the value *k*_2_ = 0 to consider populated places with any number of inhabitants.
4. *Panoramio*: Panoramio is used to find out if a particular type of location (e.g., HistoricalPlace or Forest) is relevant. When the number of photographs *N* satisfies that *N* > *k*_3_ (where *k*_3_ is a calibration parameter), we consider that the geographic feature is relevant. For the experimental tests presented in this paper we used the value *k*_3_ = 5, which was empirically adjusted with partial tests.

### 4.3 Examples

Table 2 shows examples of some descriptions generated by our method (translated into English). Example number 1 in Table 2 corresponds to a sensor that measures water level at the geographic location defined by its geographic coordinates. In this example, the query to OpenStreetMap returns a file with thousands of nodes corresponding to different labels in the bounding box around this spatial location. According to the production rules, our method searches for the name of a reservoir in this information and finds the text “Pantà de Sau” (translated as “Sau reservoir”), which is the final description.

**Table 2:** Examples of generated descriptions.

Number	Type of Measure	Latitude	Longitude	Generated Description
1	Water level	41.9697456359863	2.41436839103699	Sau reservoir
2	Rainfall	36.9890556335449	−2.35419869422913	Alhamilla Mountains
3	Water flow	38.4831809997559	0.796071231365204	River Vinalopó at Elda
4	Rainfall	40.034252166748	3.62156891822815	Aranjuez
5	Water flow	38.2390022277832	1.42935407161713	River Segura at Cieza
6	Rainfall	38.2390022277832	−1.96505701541901	San Miguel Mountains
7	Water level	38.3932685852051	−2.20556235313416	Fuensanta reservoir
8	Rainfall	38.7551918029785	−3.40385103225708	Valdepeñas

Example 2 in Table 2 corresponds to a pluviometer (type of measure: Rainfall). The method applies the production rules, and when the method searches for the feature MountainArea, it searches first in the OpenStreetMap information with the label PEAK, but this search does not provide any valid information. Then, the method uses Geonames with the label HYPSOGRAPHIC and finds the final description “Sierra de Alhamilla” (translated as “Alhamilla Mountains”).

Example 3 is a water flow sensor. In this case, our method finds the text “Río Vinalopó en Elda” (translated as “River Vinalopó at Elda”). Here, the method searches for the feature River in the OpenStreetMap information and finds “River Vinalopó”. Then, the method searches for the feature PopulatedPlace in the OpenStreetMap information and gets an ordered list of two towns near the sensor (“Elda”, “Petrer”). Our method selects the first town off the list, “Elda”, as the most appropriate place and constructs the final description “River Vinalopó at Elda” using the text template.

## 5. Evaluation Procedure

This section describes the evaluation procedure that we applied to the approach presented in this paper. Basically, we built an evaluation dataset from a real sensor network with thousands of sensor descriptions created by different domain experts. We used the accuracy as an assessment metric for the performance of our method together with a baseline accuracy to be used as comparison reference.

### 5.1. Evaluation Dataset

To evaluate the approach presented in this paper, we built an evaluation dataset using a hydrological sensor network. We used information from the Spanish national hydrological sensor network SAIH. This network has an appropriate size (thousands of sensors) to be a good representative example of the problem described in this paper.

We built the evaluation dataset using public information for the SAIH network, available on the website of the Ministry of Environment in Spain. This information is generated and maintained by the SAIH control centers at the main river basins in Spain, with the support and coordination of the Ministry of Environment. For each sensor, the dataset includes a text description written manually by domain experts. In total, there are 10 control centers with local sensor networks. Therefore, the names were written by at least 10 different experts.

**Figure 5:**
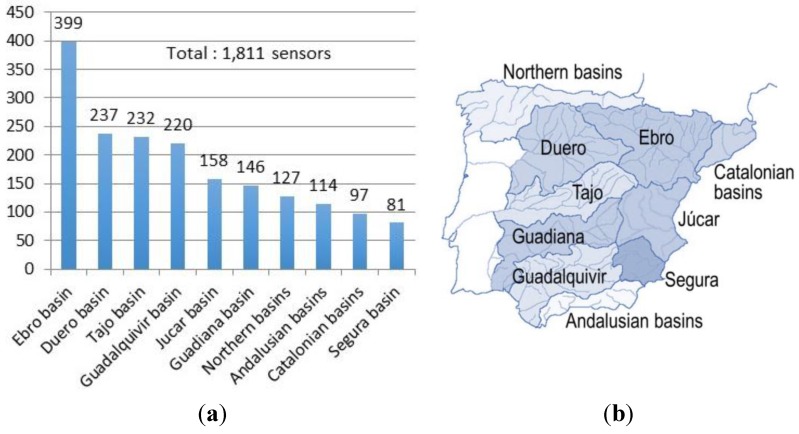
Number of sensors in the evaluation dataset for each geographic area; (**a**) number of sensors; (**b**) geographic areas.

The dataset includes a total of 1811 sensors distributed in 10 geographic areas (Figure 5). In order to build the evaluation dataset it was necessary to clean and filter the original data in order to select the appropriate information useful for our problem. For example, we removed non-useful attributes (e.g., specific identifiers or flags related to their operational status).

We provide this dataset as supplementary material for this paper. The dataset is a .csv file where each line corresponds to one sensor, and there are five attributes for each sensor:

- *Latitude* and *longitude*: Geographic coordinates of the point where the sensor is located (using the geodetic reference system is WSG84).
- *Measure*: Type of physical quantity measured by the sensor, such as water flow, rainfall, and water level.
- *Area*: The geographic area of the sensor. It is described by the name of the main river basin (e.g., Ebro basin, Duero basin, *etc.*).
- *Description*: The complete textual description written by domain experts in natural language (in Spanish) for the geographical point where the sensor is located (for example, the Ebro river at Ascó). This information is used as the gold standard to be compared to generated descriptions.

### 5.2. Baseline Accuracy

We used the evaluation dataset described in the previous section to assess the quality of descriptions generated by our method, with textual descriptions provided by domain experts. We used accuracy as an evaluation metric for the performance of our method. We applied our method to the sensors of the dataset to obtain a text description for each one. We counted the number of correct descriptions generated by our method in relation to the total number of sensors. We assumed that a description is correct when the geographic references of the description are the same as the geographic references of the description in the dataset as given by domain experts.

In order to have a baseline accuracy, we used an alternative method to generate sensor descriptions based only on the geographic database Geonames. In principle, this alternative solution is an adequate approach that has been successfully used by many applications to generate textual descriptions for geographic locations and requires a lower implementation effort.

We used the evaluation dataset to obtain the accuracy of the solution based on Geonames and obtained an accuracy of 0.30 (the baseline accuracy). The solution based on Geonames that we used to generate text descriptions for a sensor location with geographic coordinates (*x*, *y*) works as follows:

- *Pluviometer*: The textual description for a pluviometer is the name provided by Geonames for (*x*, *y*) corresponding to the label ADMINISTRATIVE.
- *Water level sensor (reservoir)*: The textual description for a water level sensor is the name provided by Geonames for (*x*, *y*) corresponding to the label HYDROGRAPHIC.
- *Water flow sensor*: The textual description for a water flow sensor is the combination of names *n*_1_ and *n*_2_ and for (*x*, *y*) corresponding respectively to the labels HYDROGRAPHIC and ADMINISTRATIVE. The description is generated as “*n*_1_ *at* *n*_2_”.

## 6. Evaluation Results

**Figure 6:**
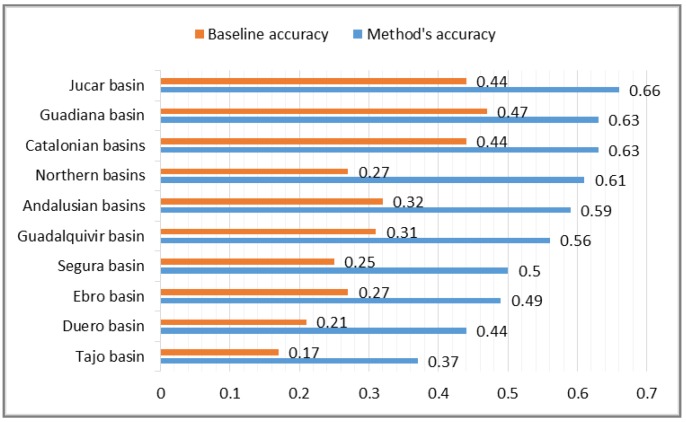
Evaluation results for geographic areas. The graphic shows the values obtained for the baseline accuracy (orange) and for our method’s accuracy (blue).

As a result of this evaluation process, we obtained a 73% improvement in accuracy with respect to the baseline. The method obtained an average accuracy of 0.52 of correct names for the complete set of sensors. There are significant differences among geographic areas corresponding to different basins (Figure 6). The best values are for the following areas: Jucar basin (0.66), Guadiana basin (0.63), and Catalonian basin (0.63). The worst value (0.37) corresponds to the Tajo basin. This low value can be justified (as it is argued later) because domain experts in this area created the descriptions following different naming strategies compared to the rest of the areas.

It is interesting to see the results according to types of measures (water level, water flow, and rainfall). For example, the average accuracy for water level obtains a good value (0.89). The three best values for water level descriptions are for the following areas: Jucar basin (1.00), Guadalquivir basin (0.93), and Ebro basin (0.92). Here, our method obtains good results when there is available information in geographic databases.

The average accuracy for water flow descriptions is 0.58. Our method here obtains very good results for certain areas: Duero basin (1.00), Andalusian basin (1.00), and Jucar basin (0.88). A particular bad case here that should be considered as an outlier corresponds to the Tajo basin with a value of accuracy of 0.10. This is a special case where the experts follow a naming strategy that is not the same as the general approach.

The descriptions for pluviometers obtain an average accuracy of 0.44. The best three values are: Catalonian basin (0.64), Guadiana basin (0.61), and Jucar basin (0.57). The descriptions here obtain a worse accuracy compared to the other types of measures. An explanation for this difference is that the descriptions for pluviometers require more diverse geographic knowledge compared to other types of sensors and this information is not always present in the open geographic databases.

**Figure 7:**
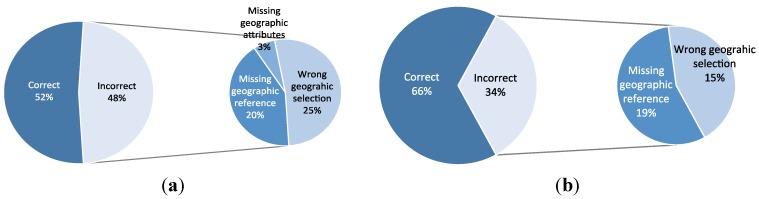
Main causes of incorrect descriptions ((**a**) average and (**b**) best geographic area).

We analyzed the main causes of the generation of incorrect descriptions. As a result, we can distinguish the following situations (Figure 7):

- *Missing geographic reference*. The required geographic reference (population, reservoir, river, mountain, monument, *etc.*) is not present in the open geographical databases. This problem happens in the 20% of the cases. The missing information could be also caused because the bounding box selected for a sensor is too small to find the correct geographic reference.
- *Missing geographic attributes*. The geographic reference is present but it is not possible to obtain some of its attributes (name of the river, number of inhabitants, *etc.*). This happens in 3% of the cases. For example, the problems can be poor information (incomplete information), overly detailed information, wrong information, or a different language.
- *Wrong geographic selection*. The correct geographic reference is present in the knowledge sources but our method wrongly selects a different reference. This happens in 25% of the cases.

In the first two situations (23% of the cases), the problems are caused by issues in the open geographic data. By solving these problems, the quality of our method could increase its performance up to a value of an average accuracy of 0.75, which means an improvement of 150% with respect to the baseline. These could be solved with more complete information or by providing different search methods (see the next section for a discussion about this issue).

In the third situation, the problems are caused by incorrect naming strategies followed by our template-based method for text generation (in 25% of the cases). To improve this, a deeper understanding about how the experts generate names may help decrease this percentage. However, a contributing factor to this percentage is also that we used different domain experts for different geographic areas: we obtained significant differences between values for different geographic areas due to wrong geographic selection (15% for the Jucar basin and 35% for the Duero Basin). According to this, the standardization of this process following uniform naming strategies for all areas could increase the performance of our method up to an accuracy of 0.85 (e.g., this is the case of Jucar basin, assuming that the problems in open geographic areas are also solved).

## 7. Discussion

This section presents a discussion about the potential improvements that could be performed to increase the accuracy of our method. The section also discusses the generality of our approach and how it can be used across different sensor networks.

### 7.1. Potential Improvements

The analysis of the evaluation results shows that the method presented in this paper can be used to automatically generate textual descriptions of sensors. In some geographic areas, the method obtains high accuracy results when compared to the descriptions provided by domain experts. However, we also identified cases with low accuracy that correspond to problems related to open geographic data. To solve such problems, we describe some possible improvements in geographic databases that would increase the global accuracy of our method up to a value of 0.75. Some improvements are related to the content of geographical databases:

- *Uniform content*. The information in open databases should present more uniformity across geographic areas. In contrast to this, the current situation is that certain geographic areas in OpenStreetMap present more detailed information than others. In certain cases, important information for our method is not present (e.g., reservoirs, historical monuments, *etc.*). In our evaluation, we found significant differences for the percentage of missing geographic references: Segura basin (20%) and Catalonian basins (10%).
- *Standard labels*. The strategies to assign labels for the stored information should be standardized, following common practices. In contrast, some information labeled in OpenStreetMap is not standard (for example, in Catalonia) and, sometimes, overly specific names are used for general names (e.g., “wall” instead of “reservoir”). The consequence of this problem is that our method is not able to find some information that is searched for, even when this information is present in databases.
- *Multilingual content*. The data should cover both local languages and international descriptions. For example, in Geonames and OpenStreetMap, a significant problem is the presence of different languages (e.g., English instead of Spanish for certain locations).

Another type of improvement is related to search methods in open geographic databases. OpenStreetMap provides a search method based on bounding boxes. This solution establishes a rigid spatial restriction and can return a large amount of non-useful information when the search is interested only in a few labels. Instead of this, it would be useful to have a more flexible search method based on labels that returns up to a maximum amount of data for specific labels around a given geographic point.

Besides these suggested improvements related to open geographic databases, we can also mention other potential extensions in our method to be able to generate richer textual expressions. For example, geographic descriptions can use locative expressions such as “near Madrid” or “west of Denver” [23]. Our method uses a few locative expressions (e.g., “the Ebro river at Ascó”). A potential improvement of our method is to include more complex locative expressions. For example, a framework for generating locative expressions is proposed by Kelleher and Kruijff [24]. A more general solution may require additional functions for spatial reasoning (distances and orientations), more sophisticated geographic locations (roads, coasts, *etc.*), approximate reasoning (e.g., fuzzy logic models), *etc.*

### 7.2. Flexibility and Generality of the Method

To analyze the flexibility and generality of the method, let us consider that the method is applied to a particular sensor network (e.g., the SAIH network) and, then, we are interested in making some changes to the network by adding more sensors. We distinguish two different scenarios of changes:

- *Add new sensors of the same type of measures*. The addition of new sensors corresponding to previously considered measures (e.g., flow, level, rain, *etc.*) does not require any change in the method. It is very important to offer this level of flexibility because, in a real sensor network such as the SAIH network, new sensors are added to or removed from network periodically (e.g., every year) as a result of maintenance procedures.
- *Add new sensors of different types of measures*. Our method needs to be changed if we want to add new sensors corresponding to different types of measures (e.g., temperature, air pollution, *etc.*). However, this is a small change that can be done easily because it only affects to the set of production rules for the textual templates. In this case, it is necessary to add new rules that relate the new type of measures and the new textual templates. This has to be done only once for the new type of measure, with a few rules, so the required effort is very low.

Therefore, the design of our method is general and independent from the sensor network and could be applied to other types of networks. The only dependence between a type of sensor measurement and a particular textual template can be established when necessary by a few production rules defined by the developer that encapsulate the way domain experts describe these measures.

## 8. Conclusions

In this paper, we have described the problem of generating text descriptions for spatially distributed sensors. A solution to this problem can help to construct more usable user interfaces for geographically distributed sensor networks.

In the paper, we have presented a method for this problem using online geographic information databases. The evaluation results show that the method generates acceptable textual descriptions. We also found that the improvement of online geographic databases could improve the accuracy of our method. According to our analysis, the geographic databases present problems related to heterogeneity, non-standard use of labels, or limited search methods.

In the paper, we have also described an evaluation dataset with thousands of sensors with text descriptions written from human experts. The evaluation dataset has been developed using information from a national hydrological sensor network in Spain. The dataset allows developers to evaluate the performance of other algorithms that automatically generate text descriptions for sensors. The analysis of the evaluation dataset (manually or automatically) is also useful to identify general strategies followed by human experts to give names to sensors.

Our current research work includes the design and development of methods to generate natural language descriptions for sensor networks. We are planning to use these methods as part of complex multimedia presentation systems that help users analyze data from large sensor networks.

## Supplementary Material

We provide the evaluation dataset used in our experiments as supplementary material for this paper. The dataset is a .csv file, corresponding to 1811 sensors with the following fields described in the paper: Latitude, longitude, measure, area, and description. Supplementary materials can be accessed at: http://www.mdpi.com/1424-8220/15/7/16009/s1.
